# Evaluation of agreement between hemoglobin A1c, fasting glucose, and fructosamine in Senegalese individuals with and without sickle-cell trait

**DOI:** 10.1371/journal.pone.0212552

**Published:** 2019-02-15

**Authors:** Sarah Skinner, Mor Diaw, Maïmouna Ndour Mbaye, Philippe Joly, Céline Renoux, Céline Masson, Charlotte Cuerq, Philomène Lopez, Malick Ndour, Demba Diedhiou, Djiby Sow, Saliou Diop, Abdoulaye Samb, Vincent Pialoux, Philippe Connes

**Affiliations:** 1 Inter-university Laboratory of Biology of Motor Function EA7424, « Vascular Biology and the Red Blood Cell » team, Claude Bernard University Lyon 1, University de Lyon 1, Villeurbanne, France; 2 Laboratory of Excellence GR-Ex, Paris, France; 3 Laboratory of Physiology and Functional Exploration, FMPO, UCAD, Dakar, Senegal; 4 Medical Clinic II, Abass Ndao Hospital Center, Dakar, Senegal; 5 Laboratory of Biochemistry of Erythrocyte Pathologies, Biology Center East, Bron, France; 6 Laboratory of Biological research on Nutrition and Diabetes, Biology Center South, Pierre-Bénite, France; 7 Laboratory of Pharmaceutical Biochemistry, University of Medicine, of Pharmacy, and Odontology, Cheikh University Anta Diop-Dakar, Senegal; 8 Laboratory of Hemato-ummunology, FMPO, UCAD, Dakar, Senegal; 9 Institute of Universities of France, Paris, France; University of Naples Federico II, ITALY

## Abstract

Fasting glucose (FG) and glycated hemoglobin A1c (HbA1c) perform sub-optimally in people of African origin, especially in individuals with sickle-cell trait (SCT). The purpose of this study was to compare the relationships between HbA1c, FG, and fructosamine in individuals from Senegal with and without SCT. HbA1c, FG, and fructosamine were measured in 203 adults from Senegal (100 control: 45 with type 2 diabetes (T2D); 103 SCT: 51 with T2D). Significant, positive correlations were observed between HbA1c and FG, fructosamine and FG, and fructosamine and HbA1c in both groups. The limits of agreement were inappropriately large in both groups for the Bland-Altman plots of HbA1c and FG (control: -95.97 to 83.97%; SCT: -115.9 to 91.52%), fructosamine and FG (control: -100.6 to 99.89%; SCT: -105.6 to 100.6%), and fructosamine and HbA1c (control: -52.03 to 38.98%; SCT: -88.04 to 71.41%). In both groups, the greatest proportion of subjects were considered above the clinical cut-point for hyperglycemia when fructosamine was used as the criterion (control: 33%; SCT: 44.6%), and the lowest percentage of subjects were classified as over the clinical cut-point when HbA1c was used as the criterion (control: 21%; SCT: 27.7%).Substantial disparities between HbA1c, FG, and fructosamine were observed in both groups, and these differences were exaggerated in the SCT group. Therefore, these three biomarkers should not be considered to be interchangeable measures of glycemic control. These biomarkers should be used thoughtfully, and special care should be taken when using them in individuals with SCT.

## Introduction

Sickle-cell trait (SCT), the heterozygous form of sickle-cell disease, is highly prevalent in Africa, especially in Central and Western Africa where between 10–30% of the population are carriers of the hemoglobin S (HbS) gene [1]. In 2017, the International Diabetes Federation (IDF) estimated that more than 15.9 million people in Africa had type 2 diabetes (T2D), and the prevalence is expected to increase 162% by 2045 [2]. This data indicates that there is likely a large, and growing, population of individuals in Africa who have both T2D and SCT.

Early diagnosis and effective monitoring of T2D are essential to decreasing the risk of developing T2D-related complications [3]. However, 70% of people in Africa who have T2D do not know that they have the disease, a higher percentage than in any other region of the world [2]. Although this problem is often attributed to a lack of access to adequate health care, inadequate performance of diagnostic tests may also contribute to this issue [3].

The oral glucose tolerance test (OGTT) is the gold standard for T2D diagnosis [3, 4]. However, the OGTT is expensive, requires patients to arrive in a fasted state, and demands a substantial time commitment from patients and medical professionals [3, 5]. For these reasons, fasting glucose (FG) and hemoglobin A1c (HbA1c) are the tests most commonly used by clinicians and epidemiologists around the globe to deliver patient care and generate public health statistics [3].

Measurement of HbA1c, a glycated form of hemoglobin A, does not require an overnight fast and reflects glucose control over a 2–3 month period [5, 6]. HbA1c has been widely used as a standard measure of glycemic control since the 1980s, and was recently recommended for use as a diagnostic test for diabetes by a committee of international experts [3, 5, 7]. However, HbA1c performs sub-optimally in people with SCT, likely due to the presence of HbS in SCT erythrocytes, thereby complicating T2D diagnosis in these individuals [8, 9]. Furthermore, FG measurement, which requires an overnight fast, has been shown to have a sensitivity of less than 50% in individuals of African decent [3]. The disadvantages associated with traditional measures of glucose tolerance have lead to an increased interest in alternative markers of hyperglycemia, like glycated proteins [6, 10].

Fructosamine is a measure of total glycated serum protein that does not require an overnight fast, is cheaper and easier to perform than HbA1c assays, and could overcome the limitations of HbA1c in certain patients with conditions that affect the reliability of HbA1c, such as SCT [5, 6]. This biomarker has a half-life of 14–21 days, and thus measures glucose control over a 2–3 week period [6]. Fructosamine is significantly associated with HbA1c and FG, and has been shown to independently predict T2D incidence and microvascular complications in populations including both black and white individuals with and without T2D [10–12]. However, the relationships between HbA1c, FG, and fructosamine have not been studied in Africans with SCT. Therefore, the objective of this study was to compare the relationships between HbA1c, FG, and fructosamine in individuals with and without SCT (with normal glycemic control and T2D) living in Senegal.

## Materials and methods

### Setting and participants

The study was conducted at the Cheikh Anta Diop University in Dakar, Senegal. A total of 203 subjects were recruited between August and October of 2017 from the Anti-Diabetic Centre (Marc Sanakalé) at the Hospital Abass Ndao in Dakar, from the National Center of Blood Transfusion (CNTS) of Dakar, and from the general population of Dakar. The study population included individuals with (n = 103; SCT) and without (n = 100; control) SCT. 45% of the control group and 49.5% of the SCT group had previously been diagnosed with T2D using the American Diabetes Association (ADA) Standards of Medical Care guidelines [4]. The large majority of the subjects with T2D (84.4% control; 84.3% SCT) in the study were being treated with metformin, sulfonylureas, or a combination of the two. Biological analyses were conducted to confirm whether or not subjects were carriers of SCT (blood was screened using isoelectric focusing, and results were confirmed using citrate agar electrophoresis, hemoglobin fraction quantification using high-performance liquid chromatography, and a solubility test confirming the presence of HbS). Hemoglobin concentrations and hematocrit were also measured in all patients. The protocol was conducted in accordance with the guidelines set by the Declaration of Helsinki and was approved by the Ethics Committee of Cheikh Anta Diop University (reference: 0221/2016/CER/UCAD). All subjects gave informed, written consent.

### Blood samples and glycemic markers

All biomarkers of hyperglycemia were measured using blood samples drawn at 8:00 am following an overnight fast. Blood was drawn into fluoride tubes for glucose measurement and EDTA tubes for HbA1c and fructosamine analyses. FG was measured using an enzymatic glucosidase-peroxydase method (Urit Medical Electronic Co., Guilin, China). HbA1c was measured using capillary electrophoresis on a Capillary 3 Tera device (Sebia, France). Fructosamine was measured in plasma using a colorimetric assay with nitrotetrazolium blue (ABX Pentra Fructosamine kit, Horiba, Montpellier, France).

### Statistical analysis

Results are presented as means ± Standard Deviation (SD). Independent Samples t-tests were performed to determine between group differences for hemoglobin concentrations, hematocrit, age, body mass index (BMI), HbA1c, FG, and fructosamine. A χ^2^ test was used to compare the sex distributions. Independent Samples t-tests were used to test for significant differences in average HbA1c, FG, and fructosamine between male and female subjects in the overall study population, and within the control and SCT groups. Pearson correlations were used to test the associations between HbA1c and FG, fructosamine and FG, and HbA1c and Fructosamine in the control and SCT groups. Additional Pearson correlations were also conducted to evaluate the associations between all of the measures of glycemic control in the subjects with T2D in the control and SCT groups, independently. Differences between the correlation coefficients of the two groups were then analyzed for statistical significance using Fisher’s *r* to *z* transformation, followed by a calculation of the observed *z*-test statistic.

The agreement between HbA1c and FG, fructosamine and FG, and HbA1c and fructosamine for each group was determined using Bland-Altman Analyses [13]. In order to compare HbA1c, FG, and fructosamine, we normalized the values by expressing them as percentages of a clinical-cut point for the diagnosis of T2D. For HbA1c and FG, we used the guidelines established by the ADA for diabetes diagnosis (HbA1c ≥6.5% and FG ≥126 mg/dL, respectively) [4]. However, clinical guidelines for T2D diagnosis using fructosamine have yet to be established. Nevertheless, a recent study conducted by Parrinello et al determined that a fructosamine value of 260 μmol/L was equivalent to a FG value of 126 mg/dL in a population of black adults [14]. Furthermore, a study conducted by Malström and colleagues concluded that it is possible to identify subjects with T2D with reasonable sensitivity and specificity using fructosamine values in the range of 240–260 μmol/L [15]. Therefore, we chose to fix the clinical cut-point for fructosamine at 260 μmol/L. For each plot, the bias was calculated as the mean difference between the two measures. To test whether the bias of each plot was significantly different from zero, a one-sample t-test was performed. The upper and lower limits of agreement (LOA) were defined as ± 1.96 SD of the mean difference. Finally a linear regression line was drawn on the Bland-Altman plot to assess possible changes in bias with decreasing glucose control.

The percentage of subjects in each group classified as over the clinical cut-point for each biomarker was determined and then compared. Chi-Square Tests were conducted to test whether the percentages of subjects classified as above the clinical cut-point for each biomarker were significantly different within each group. Furthermore, an additional analysis was carried out in which subjects with borderline HbA1c values, defined using the ADA definition of prediabetes (HbA1c between 5.7–6.4%), were identified [4]. Average HbA1c, FG, and fructosamine were then compared between the subjects identified as having prediabetes and the subjects identified as over the cut-point when HbA1c was used as the criterion.

Significance level was defined as *p*<0.05. All statistical analyses were conducted using SPSS (v.24, IBM SPSS Statistics, Chicago, IL). Bland-Altman Plots were drawn using GraphPad Prism version 6 (GraphPad, Sand Diego, CA).

## Results

### Comparisons between control and SCT groups

The descriptive characteristics of the subjects are shown in Table 1. The average FG, HbA1c, and fructosamine values for each group as well as the average percent cut-points of each value are shown in Table 2. There were no significant differences between groups for hemoglobin concentration, hematocrit age, body mass index, FG, HbA1c, or fructosamine. One subject was determined to be anemic, and was therefore excluded from the analyses. The percentage of male and female subjects did not vary significantly between the two groups. Average FG, HbA1c, and fructosamine did not vary between the male and female subjects. Additionally, analyses of the control and SCT groups individually showed that neither average HbA1c nor average fructosamine varied significantly between the male and female subjects in the control and SCT groups. Average FG was higher in the female subjects (132.61 ± 68.80 mg/dL) compared to the male subjects (104.36 ± 35.58 mg/dL) in the control group only (*p* = 0.01).

**Table 1:** Participant characteristics.

	Control	SCT
Total (n =)	100	103
Sex (M/F)	25/75	16/87
Hemoglobin concentration (g/dL) (M/F)	12.87 ± 1.52/12.63 ± 1.83	12.42 ± 2.22/12.59 ± 1.38
Hematocrit (%)	40.06 ± 2.4	40.05 ± 2.4
T2D	45 (45.00%)	51 (49.51%)
Age (years)	51.90 ± 7.64	50.79 ± 7.60
BMI (kg/m2)	24.81 ± 4.27	24.65 ± 4.09

T2D = Type 2 diabetes

BMI = body mass index; HbS = hemoglobin S

Results are presented as means ± SD. No significant differences were found between groups.

**Table 2:** Biomarkers of hyperglycemia by group.

	Control		SCT	
	Original value	% Cut-point (%)	Original value	% Cut-point (%)
FG	125.69 ± 62.86 mg/dL	99.75 ± 49.89	139.73 ± 77.79 mg/dL	110.9 ± 61.73
HbA1c	6.03 ± 1.55%	89.15 ± 29.72	6.54 ± 2.31%	95.81 ± 40.92
Fructosamine	259.38 ± 86.80 μmol/L	96.76 ± 37.05	283.09 ± 128.05 μmol/L	107.00 ± 51.24

Average values of each biomarker are expressed as whole values and as normalized values, which were calculated as percentages of a clinical cut-point. Clinical cut-points were set at 126 mg/dL for FG, 6.5% for HbA1c, and 260 μmol for fructosamine.

FG = Fasting Glucose; HbA1c = Glycated Hemoglobin A

Results are presented as means ± SD. No significant differences were found between groups.

### Correlations

**Fig 1:**
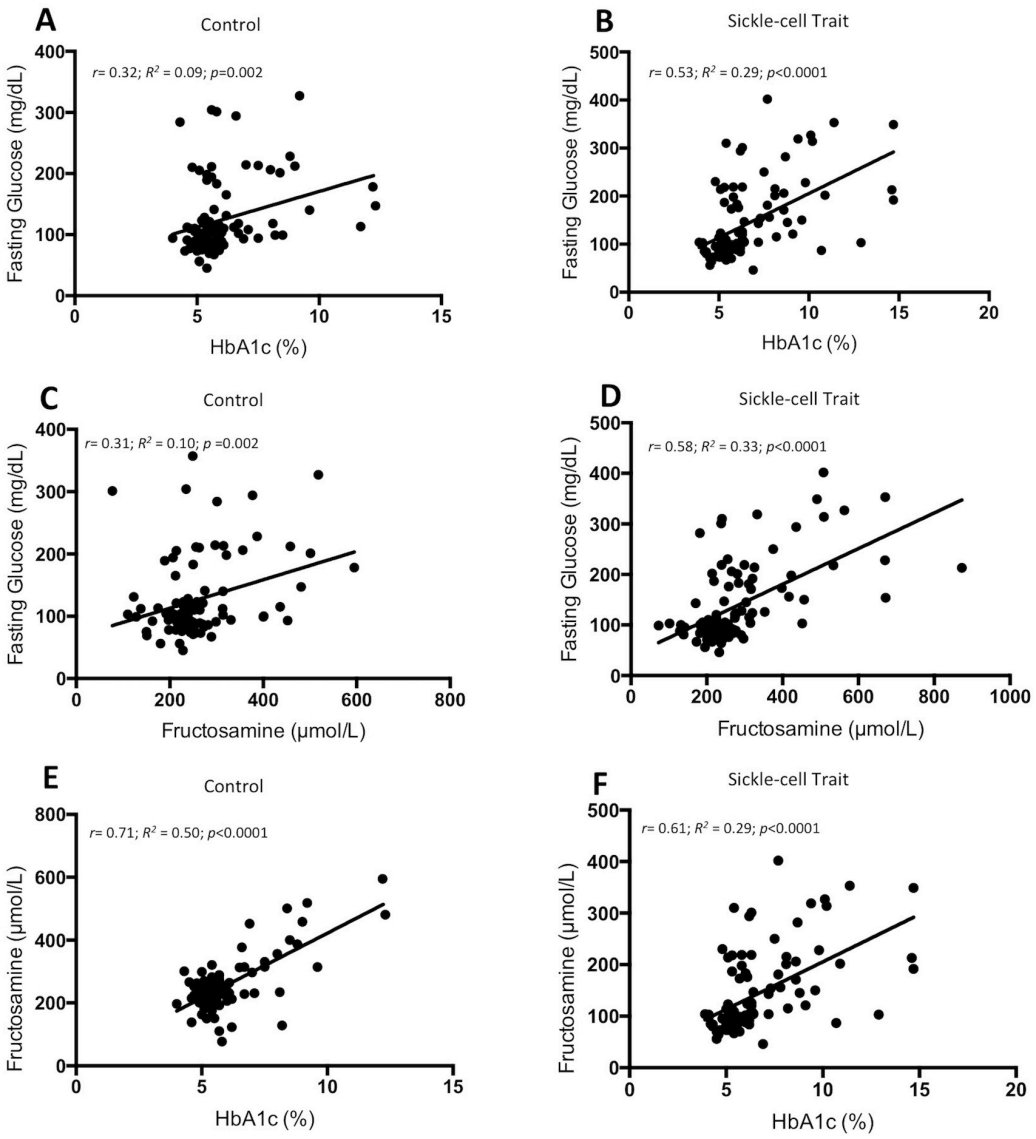
Scatterplots of glycated hemoglobin A1c (HbA1c) versus fasting glucose (FG), fructosamine versus FG, and HbA1c versus fructosamine for control and SCT groups. The best-fit model (solid line) is shown for each plot along with the coefficient of determination (R^2^), the Pearson correlation coefficient, and the p-value of the correlation.

HbA1c and FG, and fructosamine and FG were weakly correlated in the control group (r = 0.32; *p* = 0.002 and r = 0.31; *p* = 0.002, respectively) and moderately correlated in the SCT group (r = 0.53; *p* = <0.0001 and r = 0.58; *p*<0.0001, respectively). The correlations between fructosamine and HbA1c were fairly strong in both the control and SCT groups (r = 0.71; *p*<0.0001 and r = 0.61; *p*<0.0001, respectively) (Fig 1). The correlation between fructosamine and FG was significantly stronger in the SCT group compared to the control group (*p*<0.05). The strength of the correlations did not differ between the groups for the correlations between HbA1c and FG (*p =* 0.07) or fructosamine and HbA1c (*p =* 0.21).

When subjects with T2D in the control and SCT groups were considered independently, HbA1c and fructosamine were significantly correlated in the subjects with T2D in the control and SCT groups (r = 0. 68; *p*<0.0001 and r = 0.48; *p*<0.0001, respectively). Fructosamine and FG were significantly correlated in the subjects with T2D in the SCT group (r = 0.39; *p*<0.01), but not in the control group. HbA1c and FG were not significantly correlated in the subjects with T2D in the control or SCT groups.

### Bland-Altman analyses

**Fig 2:**
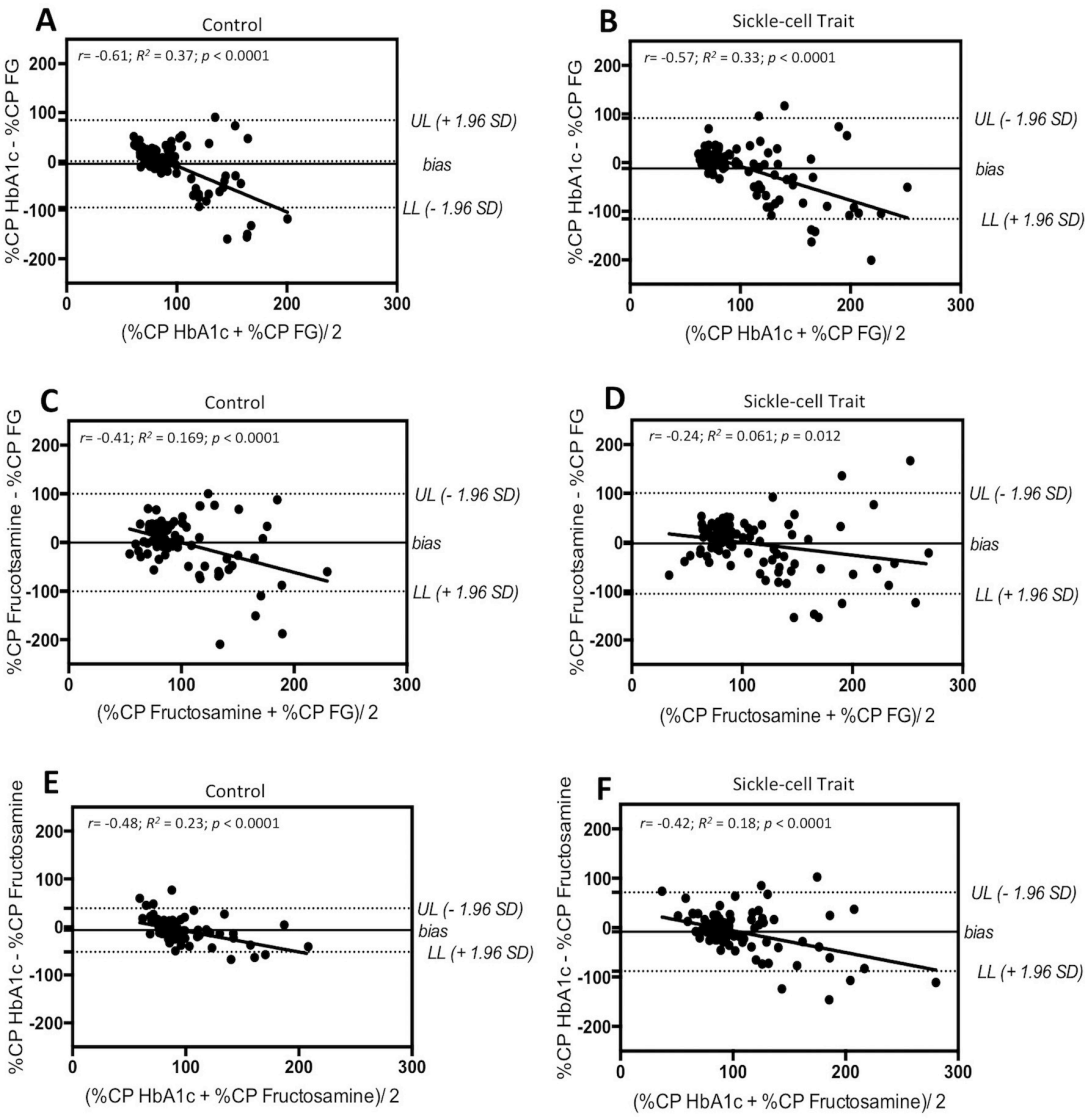
Bland-Altman plots showing the agreement between glycated hemoglobin A1c (HbA1c) and fasting glucose (FG) (plots A and B), fructosamine and FG (plots C and D), and HbA1c and fructosamine (plots E and F) for the control (plots A, C, and E) and sickle-cell trait groups (plots B, D, F). Each plot represents the mean of the two methods (x-axis) against their differences. The mean difference between the two methods (bias) is shown, as are the Upper (UL) and Lower limits (LL) of agreement. %CP HbA1c = percent of the clinical cut-point of HbA1c. %CP FG = percent of the clinical cut-point of fasting glucose. %CP Fructosamine = percent of the clinical cut-point of fructosamine.

The results of the Bland-Altman analyses are displayed in Fig 2.

#### HbA1c and FG

The mean biases revealed that the FG values were on average 5.54 ± 45.67% (95% LOA from -95.97 to 83.97%) and 12.21 ± 52.92% (95% LOA from -115.9 to 91.52%) greater than the HbA1c values in the control and SCT groups, respectively. One-sample t-tests showed that the bias was significantly different from zero in the SCT group (*p* = 0.02), but not in the control group (*p* = 0.24). The percentage of dots outside the LOA was relatively low in both groups (control: 6.2%; SCT: 6.1%). However, the LOA were unacceptably large for both groups. Thus, the two measures cannot be considered to be interchangeable. Furthermore, both plots show a moderate tendency for the bias to become negative, and more variable, with increasing glycemia in both groups (Control: *r* = -0.61; *R*^*2*^ = 0.37; *p*<0.0001 and SCT: *r* = -0.57; *R*^*2*^ = 0.33; *p*<0.0001).

#### Fructosamine and FG

The mean biases showed that the FG values were on average 0.37 ± 51.16% (95% LOA from -100.6 to 99.89%) and 2.50 ± 52.59% (95% LOA from -105.6 to 100.6%) greater than the fructosamine values for the control and SCT groups, respectively. One-sample t-tests revealed that these biases were not significantly different from zero for either group (control: *p* = 0.94; SCT: *p* = 0.63). A relatively small percentage of the dots (5.1% of the control group and 6.8% of the SCT group) were outside of the LOA for both groups. However, the LOA were unacceptably large for both groups, indicating that the two measures are not interchangeable. Finally, the significant negative linear regressions show a moderate tendency for the bias to decrease with increasing glycemia in the control (*r* = -0.41; *R*^*2*^ = 0.169; *p*<0.0001) and SCT (*r* = -0.24; *R*^*2*^ = 0.061; *p* = 0.012) groups. Both plots showed that variability tended to increase in both groups as glycemia increased.

#### Fructosamine and HbA1c

The mean biases showed that the fructosamine values were on average 6.52 ± 23.22% (95% LOA from -52.03 to 38.98%) and 8.31 ± 40.68% (95% LOA from -88.04 to 71.41%) larger than the HbA1c values for the control and SCT groups, respectively. The one-sample t-tests demonstrated that the biases for the two groups were significantly different from zero (control: *p* = 0.008; SCT: *p* = 0.046). The percentage of dots outside of the LOA was fairly small and similar between the two groups (7.5% of the control group and 8.2% of the SCT group). The LOA were unacceptably large for both groups, but they were much larger in the SCT than in the control group. This suggests that HbA1c and fructosamine are not interchangeable, especially not for carriers of SCT. Additionally, the negative linear regressions show a moderate tendency for the bias to become more negative and more variable as glycemia increases in both groups (Control: *r* = -0.48; *R*^*2*^ = 0.23; *p*<0.0001 and SCT: *r* = -0.42; *R*^*2*^ = 0.18; *p*<0.0001).

### Classification as over the clinical cut-point and chi-square analyses

10% more control subjects (four with T2D) and 12.1% more SCT subjects (eleven with T2D) were considered over the clinical cut-point when classified using FG as the criterion versus HbA1c. The chi-square analyses showed that the number of subjects classified as over the clinical cut-point was significantly greater when using FG as compared to HbA1c in both the control (*p* = 0.016) and SCT (*p*<0.0001) groups.

2% more control (neither diagnosed with T2D) and 4.8% more SCT (none with T2D) subjects were classified as above the clinical cut-point when using fructosamine rather than FG as the glycemic parameter. The chi-square analyses showed that the proportion of subjects classified as above the clinical cut-point when using fructosamine as the criterion was significantly greater than when using FG in both the control (*p* = 0.003) and SCT (*p*<0.0001) groups.

12% more control (four with T2D) and 16.9% more SCT subjects (eight with T2D) were considered above the clinical cut-point when classified using fructosamine versus HbA1c. The results of the chi-square analyses indicated that these differences were significantly different in both the control (*p*<0.0001) and SCT (*p*<0.0001) groups (Table 3).

**Table 3:** Number of subjects classified as over the clinical cut-point for each biomarker of hyperglycemia.

	FG	HbA1c	Fructosamine
Control	31 (31.0%)	21 (21.0%)	33 (33%)
SCT	41 (39.8%)	28 (27.7%)	46 (44.6%)

Clinical cut-points were set at 126 mg/dL for FG, 6.5% for HbA1c, and 260 μmol for fructosamine.

FG = Fasting Glucose; HbA1c = Glycated Hemoglobin

Average HbA1c and fructosamine values were significantly lower in individuals classified as having prediabetes compared to those classified as over the cut-point in both the control and SCT groups. Fasting glucose tended to be lower (*p* = 0.06) in individuals with prediabetes compared to those classified as over the cut-point in the control group, and was significantly lower in individuals with prediabetes compared to those above the clinical cut-point in the SCT group (Table 4).

**Table 4:** Average fasting glucose, HbA1c, and fructosamine in subjects classified as having prediabetes and subjects classified as over the clinical cut point in the control and SCT groups.

	Control		SCT	
	Prediabetes	Over the cut-point	Prediabetes	Over the cut-point
n =	22	21	23	28
FG mg/dL	125.55 ± 56.86	163.20 ± 70.40	141.43 ± 67.88	204.30 ± 92.43*
HbA1c %	5.90 ± 0.19	8.21 ± 1.58*	6.07 ± 0. 25	9.33 ± 2.12*
Fructosamine μmol/L	223.76 ± 55.64	355.21 ± 114.84*	263.35 ± 83.81	375.92 ± 152.12*

Prediabetes defined as a HbA1c between 5.7–6.4%. The clinical cut-point for HbA1c (6.5%) was used.

FG = Fasting Glucose; HbA1c = Glycated Hemoglobin A

Results are presented as means ± SD

*Significantly different from prediabetes (*p*<0.05)

## Discussion

The primary finding of the study is that although HbA1c, FG, and fructosamine were significantly and positively correlated in both the control and SCT groups, analyses of agreement revealed considerable disparity between the three biomarkers of hyperglycemia. Furthermore, these differences were particularly evident in the SCT group. These results suggest that these biomarkers are not interchangeable, and should be interpreted carefully, especially in individuals with SCT.

The correlational analyses showed moderate associations between HbA1c and FG and between fructosamine and FG, as well as fairly strong associations between HbA1c and fructosamine in both groups. Additional analyses showed that HbA1c and fructosamine were significantly associated in individuals with diabetes in both groups. These findings correspond with multiple studies that have found significant, positive associations between HbA1c, fructosamine, and FG in individuals with and without diabetes [5, 15–18]. However, our findings that fructosamine and FG were associated in people with diabetes in the SCT group only, while HbA1c and FG were not associated in subjects with diabetes in either group, conflict with these previous findings [5, 15–18].

Furthermore, our results coincide with the findings of a study by Jurascheck et al, which also observed a stronger correlation between fructosamine and HbA1c than fructosamine and FG or HbA1c and FG [12]. In addition, as has been observed in other studies, none of the biomarkers of glycemia were very strongly correlated in the current study [5]. This could be due to the physiological differences between the three biomarkers (such as the differences in half life of HbA1c and fructosamine), as well as other sources of biological and analytical variability [5, 6].

Correlation analysis evaluates the linear correlation between two measures, and does not automatically imply good agreement. For this reason, Bland-Altman plots were used to interpret the bias, data scatter, and agreement between HbA1c and FG, fructosamine and FG, and HbA1c and fructosamine for both groups. The Bland-Altman analyses revealed that the LOA between all of the measures were unacceptably large in both groups. Interestingly, the LOA tended to be larger in the SCT group compared to the control group. This phenomenon was particularly evident in the comparisons of HbA1c and fructosamine. Overall, the analyses of agreement revealed considerable disparities between the three biomarkers of hyperglycemia, and these differences were heightened in the SCT group. These disparities suggest that these biomarkers should not be considered to be interchangeable measures of hyperglycemia. Use of only one of these biomarkers could possibly result in an erroneous assessment of blood glucose control, potentially leading to missed diagnoses or inappropriate follow-up of T2D. Furthermore, there was a moderate tendency for the bias to become more negative, and more variable, as glycemic control became worse. These results indicate that the disparities between the three measures may be the most pronounced in the patients who have the poorest glycemic control.

Variability between HbA1c and other markers of glycemia has been widely reported, especially in populations of African decent [19–21]. For example, a study conducted by Bergenstal and colleagues in 2017 concluded that HbA1c overestimates average glucose in black individuals versus white individuals, likely because of racial differences in hemoglobin glycation [19]. However, accumulating evidence suggests that hemoglobin glycation could be more heavily influenced by inter-individual variability than by race or ethnicity, due to a variety of factors like differences in extra- and intra-cellular glucose balance, non-glycemic genetic determinants of hemoglobin glycation, and red blood cell survival [20, 22]. Additionally, evidence shows that HbA1c can be affected by sex, however no differences in average HbA1c were observed between the male and female subjects in the control or SCT groups in this study [23].

Disparities between HbA1c and fructosamine have been observed in several other studies [16–18, 24]. Indeed, Macdonald et al compared measured HbA1c to estimated HbA1c from fructosamine values in 1744 patients and found a substantial difference between the two measures, leading to the conclusion that HbA1c may not accurately reflect glucose control [24]. Similarly, Narbonne et al found that estimating HbA1c from fructosamine values did not provide an accurate enough measure of glucose control to evaluate the efficacy of diabetic treatments [17]. Furthermore, studies conducted by Nayak et al and Cohen et al have shown that the discordance between HbA1c and fructosamine, referred to as the glycation gap, remains consistent within subjects and is positively associated with nephropathy, a common diabetes-related complication [18]. These studies suggest that the consistent disparities between HbA1c (a measure of intracellular glycation) and fructosamine (a measure of extra-cellular glycemic control) could be relevant to the pathophysiology of diabetes-related vascular complications [16, 18]. A growing body of research shows that SCT increases the risk of chronic kidney disease and end stage renal disease [25, 26]. Additionally, a recent study by Diaw et al found that subjects with SCT and T2D had a higher frequency of microalbuminuria compared to control subjects and subjects with SCT or T2D [27]. More studies should be done to determine whether the exaggerated discrepancies between HbA1c and fructosamine observed in the SCT group could be linked to an increased risk of nephropathy.

The lack of agreement between the three biomarkers of hyperglycemia, which was more pronounced in the SCT group, was reflected by the inconsistency in the number of subjects classified as above the clinical cut-point depending on the criterion used. For example, when HbA1c was used as the criterion, fewer subjects were classified as above the clinical cut-point than when FG or fructosamine were used, and the differences were larger in the SCT group. These results are in accordance with a study by Guo et al, which concluded that the accuracy of HbA1c varied among subjects, resulting in many missed diagnoses when used as a sole diagnostic criterion [21]. Multiple studies have suggested that using multiple biomarkers of hyperglycemia may be the best way to improve the accuracy of blood glucose monitoring and T2D diagnosis [5, 6, 22, 28, 29]. More research should be performed to understand the potential benefits of using multiple biomarkers to measure glycemic control in people with SCT.

The inclusion of individuals with T2D was a strength of the present study as it enabled us to evaluate the relationships between the three biomarkers over a large range of glucose control. Another strength of the study was that HbA1c was measured using the Sebia Capillarys 3 Tera device, a method that shows no interference from HbS, or other common hemoglobin variants according to the National Glycohemoglobin Standardization Program (NGSP) [30]. However, it is worth recognizing that the findings of Lacy et al suggest that HbA1c may consistently underestimate glycemic control in black individuals with SCT [31]. Additionally, a recent study reported that the number of functional alpha-genes in individuals with alpha-thalassemia could affect HbA1c values [32]. In this study, subjects were not tested for alpha-thalassemia. Therefore, future studies could be conducted to study the performance of HbA1c, fructosamine, and FG in individuals with SCT and alpha-thalassemia.

## Conclusions

Overall, this study showed that HbA1c, FG, and fructosamine were positively correlated in Senegalese individuals with and without SCT. However, the disparities between the three biomarkers were substantial, and were especially pronounced in the SCT group. These results suggest that the three biomarkers should not be used interchangeably to measure glycemic control, especially not in individuals with SCT. Future studies should be conducted in African individuals with and without SCT to compare the prognostic sensitivity of these biomarkers against the gold standard for diabetes diagnosis, the OGTT. Additionally, more research should be done to determine whether using multiple biomarkers could help provide complementary information about the risk of developing T2D complications, or improve the accuracy of assessments of glycemic control, thereby enabling better patient care in Africans with and without SCT.

## References

[1] WHO. Sickle-Cell Disease: A Strategy for the WHO African Region. Malabo, Equatorial Guinea: World Health Organization Regional Committee for Africa; 2010.

[2] Ogurtsova K, da Rocha Fernandes JD, Huang Y, Linnenkamp U, Guariguata L, Cho NH, et al IDF Diabetes Atlas: Global estimates for the prevalence of diabetes for 2015 and 2040. Diabetes Res Clin Pract. 2017;128:40–50. 10.1016/j.diabres.2017.03.024 28437734

[3] Utumatwishima JN, Chung ST, Bentley AR, Udahogora M, Sumner AE. Reversing the tide—diagnosis and prevention of T2DM in populations of African descent. Nat Rev Endocrinol. 2018;14(1):45–56. 10.1038/nrendo.2017.127 29052590

[4] American Diabetes A. 2. Classification and Diagnosis of Diabetes: Standards of Medical Care in Diabetes-2018. Diabetes Care. 2018;41(Suppl 1):S13–S27. 10.2337/dc18-S002 29222373

[5] Parrinello CM, Selvin E. Beyond HbA1c and glucose: the role of nontraditional glycemic markers in diabetes diagnosis, prognosis, and management. Current diabetes reports. 2014;14(11):548 10.1007/s11892-014-0548-3 25249070 PMC4214073

[6] Danese E, Montagnana M, Nouvenne A, Lippi G. Advantages and pitfalls of fructosamine and glycated albumin in the diagnosis and treatment of diabetes. J Diabetes Sci Technol. 2015;9(2):169–76. 10.1177/1932296814567227 25591856 PMC4604592

[7] International Expert C. International Expert Committee report on the role of the A1C assay in the diagnosis of diabetes. Diabetes Care. 2009;32(7):1327–34. 10.2337/dc09-9033 19502545 PMC2699715

[8] Little RR, Rohlfing CL, Sacks DB, National Glycohemoglobin Standardization Program Steering C. Status of hemoglobin A1c measurement and goals for improvement: from chaos to order for improving diabetes care. Clin Chem. 2011;57(2):205–14. 10.1373/clinchem.2010.148841 21148304

[9] Skinner S, Pialoux V, Fromy B, Sigaudo-Roussel D, Connes P. Sickle-cell trait and diagnosis of type 2 diabetes. Lancet Diabetes Endocrinol. 2018. 10.1016/S2213-8587(18)30033-0 29459113

[10] Selvin E R AM; Grams M; Klein R; Sharrett AR; Steffes M; Coresh J. Prognostic utility of fructosamine and glycated albumin for incident diabetes and microvascular complications. Lancet Diabetes Endocrinol. 2014;2(4):279–88. 10.1016/S2213-8587(13)70199-2 24703046 PMC4212648

[11] Juraschek SP, Steffes MW, Miller ER 3rd, Selvin E. Alternative markers of hyperglycemia and risk of diabetes. Diabetes Care. 2012;35(11):2265–70. 10.2337/dc12-0787 22875225 PMC3476908

[12] Juraschek SP, Steffes MW, Selvin E. Associations of alternative markers of glycemia with hemoglobin A(1c) and fasting glucose. Clin Chem. 2012;58(12):1648–55. 10.1373/clinchem.2012.188367 23019309 PMC3652236

[13] Bland JM, Altman DG. Statistical methods for assessing agreement between two methods of clinical measurement. Lancet. 1986;1(8476):307–10. 2868172

[14] Parrinello CM, Sharrett AR, Maruthur NM, Bergenstal RM, Grams ME, Coresh J, et al Racial Differences in and Prognostic Value of Biomarkers of Hyperglycemia. Diabetes Care. 2016;39(4):589–95. 10.2337/dc15-1360 26681712 PMC4806772

[15] Malmstrom H, Walldius G, Grill V, Jungner I, Gudbjornsdottir S, Hammar N. Fructosamine is a useful indicator of hyperglycaemia and glucose control in clinical and epidemiological studies--cross-sectional and longitudinal experience from the AMORIS cohort. PLoS One. 2014;9(10):e111463 10.1371/journal.pone.0111463 25353659 PMC4213035

[16] Cohen RM, Holmes YR, Chenier TC, Joiner CH. Discordance between HbA1c and fructosamine: evidence for a glycosylation gap and its relation to diabetic nephropathy. Diabetes Care. 2003;26(1):163–7. 12502674 10.2337/diacare.26.1.163

[17] Narbonne H, Renacco E, Pradel V, Portugal H, Vialettes B. Can fructosamine be a surrogate for HbA(1c) in evaluating the achievement of therapeutic goals in diabetes? Diabetes Metab. 2001;27(5 Pt 1):598–603. 11694860

[18] Nayak AU, Holland MR, Macdonald DR, Nevill A, Singh BM. Evidence for consistency of the glycation gap in diabetes. Diabetes Care. 2011;34(8):1712–6. 10.2337/dc10-1767 21715524 PMC3142043

[19] Bergenstal RM, Gal RL, Connor CG, Gubitosi-Klug R, Kruger D, Olson BA, et al Racial Differences in the Relationship of Glucose Concentrations and Hemoglobin A1c Levels. Ann Intern Med. 2017;167(2):95–102. 10.7326/M16-2596 28605777

[20] Selvin E, Sacks DB. Variability in the Relationship of Hemoglobin A1c and Average Glucose Concentrations: How Much Does Race Matter? Ann Intern Med. 2017;167(2):131–2. 10.7326/M17-1231 28605752

[21] Guo F, Moellering DR, Garvey WT. *Use of HbA1c for Diagnoses of Diabetes and Prediabetes*: *Comparison with Diagnoses Based on Fasting and 2-Hr Glucose Values and Effects of Gender*, *Race*, *and Age*. METABOLIC SYNDROME AND RELATED DISORDERS, 2014 12(5): p. 258–268. 24512556 10.1089/met.2013.0128 PMC4088353

[22] Herman WH, Cohen RM. Racial and ethnic differences in the relationship between HbA1c and blood glucose: implications for the diagnosis of diabetes. J Clin Endocrinol Metab. 2012;97(4):1067–72. 10.1210/jc.2011-1894 22238408 PMC3319188

[23] Bae JC, Suh S, Jin SM, Kim SW, Hur KY, Kim JH, et al Hemoglobin A1c values are affected by hemoglobin level and gender in non-anemic Koreans. Journal of diabetes investigation. 2014 1;5(1):60–5. 10.1111/jdi.12123 24843738 PMC4025240

[24] Macdonald DR, Hanson AM, Holland MR, Singh BM. Clinical impact of variability in HbA1c as assessed by simultaneously measuring fructosamine and use of error grid analysis. Ann Clin Biochem. 2008;45(Pt 4):421–5. 10.1258/acb.2008.007259 18583630

[25] Naik RP, Haywood C Jr. Sickle cell trait diagnosis: clinical and social implications. Hematology Am Soc Hematol Educ Program. 2015;2015:160–7. 10.1182/asheducation-2015.1.160 26637716 PMC4697437

[26] Naik RP, Irvin MR, Judd S, Gutierrez OM, Zakai NA, Derebail VK, et al Sickle Cell Trait and the Risk of ESRD in Blacks. J Am Soc Nephrol. 2017;28(7):2180–7. 10.1681/ASN.2016101086 28280138 PMC5491293

[27] Diaw M, Pialoux V, Martin C, Samb A, Diop S, Faes C, et al Sickle Cell Trait Worsens Oxidative Stress, Abnormal Blood Rheology, and Vascular Dysfunction in Type 2 Diabetes. Diabetes Care. 2015;38(11):2120–7. 10.2337/dc15-0699 26324331 PMC4613921

[28] Sumner AE, Duong MT, Aldana PC, Ricks M, Tulloch-Reid MK, Lozier JN, et al A1C Combined With Glycated Albumin Improves Detection of Prediabetes in Africans: The Africans in America Study. Diabetes Care. 2016;39(2):271–7. 10.2337/dc15-1699 26681716 PMC4876771

[29] Sumner AE, Thoreson CK, O’Connor MY, Ricks M, Chung ST, Tulloch-Reid MK, et al Detection of abnormal glucose tolerance in Africans is improved by combining A1C with fasting glucose: the Africans in America Study. Diabetes Care. 2015;38(2):213–9. 10.2337/dc14-1179 25338926 PMC4302255

[30] NGSP. HbA1c Assay Interferences: The National Hemoglobin Standardization Program (NGSP); 2016 [updated December 2016. http://www.ngsp.org/interf.asp.

[31] Lacy ME, Wellenius GA, Sumner AE, Correa A, Carnethon MR, Liem RI, et al Association of Sickle Cell Trait With Hemoglobin A1c in African Americans. JAMA. 2017;317(5):507–15. 10.1001/jama.2016.21035 28170479 PMC5713881

[32] Xu A, Ji L, Chen W, Xia Y, Zhou Y. Effects of α-thalassemia on Hba1c Measurement. Journal of clinical laboratory analysis. 2016 11;30(6):1078–80. 10.1002/jcla.21983 27184351 PMC6807009

